# Supplementary material for: Mesothelial Plasticity Specifies Divergent Pleural Immune Circuits that Instruct Lung Regeneration versus Degeneration
Source: Res Sq. 2026 Jul 31:rs.3.rs-10472664. Preprint. [Version 1] doi: 10.21203/rs.3.rs-10472664/v1 (PMC13484444; doi:10.21203/rs.3.rs-10472664/v1)
Supplement: Supplementary file 1 [file NIHPPRS10472664V1-supplement-1.pdf]

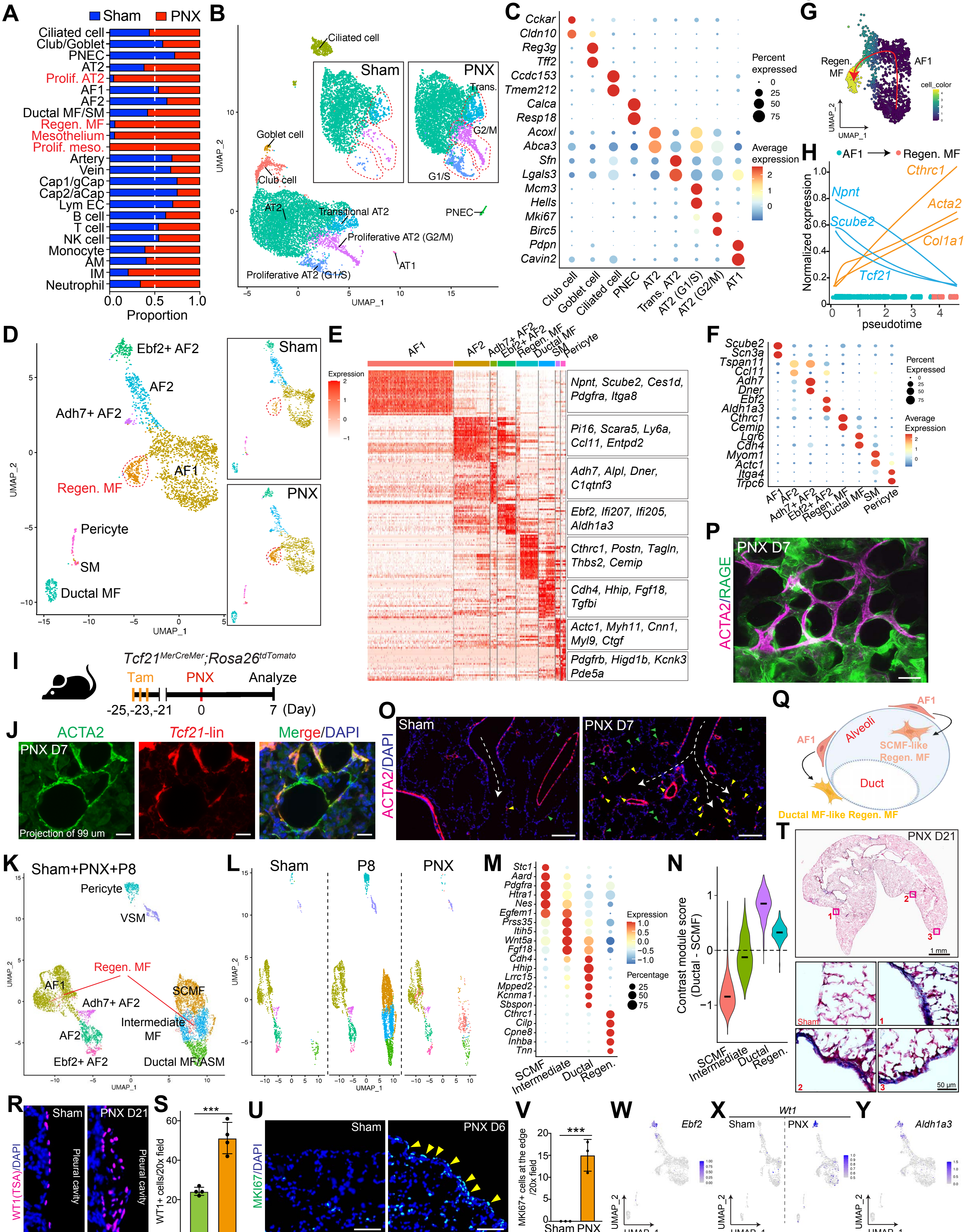

A
Sham
PNX
Ciliated cell
Club/Goblet
PNEC
AT2
Prolif. AT2
AF1
AF2
Ductal MF/SM
Regen. MF
Mesothelium
Prolif. meso.
Artery
Vein
Cap1/gCap
Cap2/aCap
Lym EC
B cell
T cell
NK cell
Monocyte
AM
IM
Neutrophil
0.0 0.5 1.0
Proportion
B
Ciliated cell
Sham
PNX
Trans.
G2/M
G1/S
Goblet cell
Club cell
AT2
Transitional AT2
PNEC
Proliferative AT2 (G2/M)
AT1
Proliferative AT2 (G1/S)
UMAP_1
UMAP_2
C
Cckar
Cldn10
Reg3g
Tff2
Ccdc153
Tmem212
Calca
Resp18
Acoxl
Abca3
Sfn
Lgals3
Mcm3
Hells
Mki67
Birc5
Pdpn
Cavin2
Percent expressed
0
25
50
75
Average expression
2
1
0
Club cell
Goblet cell
Ciliated cell
PNEC
AT2
Trans. AT2
AT2 (G1/S)
AT2 (G2/M)
AT1
G
Regen. MF
AF1
cell_color
UMAP_1
UMAP_2
H
AF1 → Regen. MF
Cthrc1
Npnt
Acta2
Scube2
Col1a1
Tcf21
Normalized expression
pseudotime
D
Ebf2+ AF2
AF2
Adh7+ AF2
AF1
Regen. MF
Pericyte
SM
Ductal MF
Sham
PNX
UMAP_1
UMAP_2
E
AF1
AF2
Adh7+ AF2
Ebf2+ AF2
Regen. MF
Ductal MF
SM
Pericyte
Expression
Npnt, Scube2, Ces1d, Pdgfra, Itga8
Pi16, Scara5, Ly6a, Ccl11, Entpd2
Adh7, Alpl, Dner, C1qtnf3
Ebf2, Ifi207, Ifi205, Aldh1a3
Cthrc1, Postn, Tagln, Thbs2, Cemip
Cdh4, Hhip, Fgf18, Tgfbi
Actc1, Myh11, Cnn1, Myl9, Ctgf
Pdgfrb, Higd1b, Kcnk3 Pde5a
F
Scube2
Scn3a
Tspan11
Ccl11
Adh7
Dner
Ebf2
Aldh1a3
Cthrc1
Cemip
Lgr6
Cdh4
Myom1
Actc1
Itga4
Trpc6
Percent Expressed
Average Expression
AF1
AF2
Adh7+ AF2
Ebf2+ AF2
Regen. MF
Ductal MF
SM
Pericyte
P
PNX D7
ACTA2/RAGE
I
Tcf21MerCreMer;Rosa26tdTomato
Tam
PNX
Analyze
-25,-23,-21
0
7 (Day)
J
ACTA2
Tcf21-lin
Merge/DAPI
PNX D7
Projection of 99 um
O
ACTA2/DAPI
Sham
PNX D7
Q
AF1
Alveoli
AF1
SCMF-like Regen. MF
Duct
Ductal MF-like Regen. MF
T
PNX D21
1 mm
Sham
50 µm
K
Sham+PNX+P8
Pericyte
VSM
Regen. MF
AF1
SCMF
Adh7+ AF2
Intermediate MF
AF2
Ebf2+ AF2
Ductal MF/ASM
UMAP_1
UMAP_2
L
Sham
P8
PNX
UMAP_1
UMAP_2
M
Stc1
Aard
Pdgfra
Htra1
Nes
Egfem1
Prss35
Itih5
Wnt5a
Fgf18
Cdh4
Hhip
Lrrc15
Mpped2
Kcnma1
Sbspon
Cthrc1
Cilp
Cpne8
Inhba
Tnn
Expression
Percentage
SCMF
Intermediate
Ductal
Regen.
N
Contrast module score (Ductal - SCMF)
SCMF
Intermediate
Ductal
Regen.
R
WT1(TSA)/DAPI
Sham
PNX D21
Pleural cavity
Pleural cavity
25 µm
S
***
WT1+ cells/20x field
Sham
PNX
U
MKI67/DAPI
Sham
PNX D6
V
***
MKI67+ cells at the edge /20x field
Sham
PNX
W
Ebf2
UMAP_1
UMAP_2
X
Wt1
Sham
PNX
UMAP_1
UMAP_2
Y
Aldh1a3
UMAP_1
UMAP_2

Figure S1. Characterization of epithelial and mesenchymal cells during lung regeneration post PNX. Related to Figure 1.

(A) Ratio of normalized cell numbers from sham and PNX lungs for each cell type.

(B) UMAP embedding of integrated epithelial cells from sham and PNX lungs at day 7 post surgery. AT2-related cells are separated into sham and PNX conditions in the boxes.

(C) Dot plot profiling of marker genes of each cell type.

(D) UMAP embedding of integrated mesenchymal cells from sham and PNX lungs at day 7 post surgery.

(E-F) Profiling of marker genes in each mesenchymal cell type, as presented by the heatmap. Representative genes were listed in the box at right. Dot plots of selected genes were shown in (F).
(G) Differentiation trajectory of regenerative MF, as presented by pseudotime analysis from Monocle 3.

(H) Expression changes of representative genes along the differentiation pseudotime.

(I) Schematic of experimental procedure used to assay cellular origin of regenerative MF post PNX in *Tcf21MerCreMer;tdT*omato mice.

(J) Representative images of ACTA2 staining with lineage marker tdTomato at day 7 post PNX. Scale bars, 20 μm.

(K-L) UMAP embedding of integrated mesenchymal cells from sham, PNX and postnatal day 8 lungs. Cells from different conditions were separately shown in (L).

(M) Dot plot profiling of marker genes of each myofibroblast-featured cell type.

(N) Quantification of SCMF- or Ductal MF-biased cell states in each cell type, as presented by contrast module scores of SCMF- and Ductal MF-related gene sets.

(O) Immunostaining of ACTA2 in sham and PNX lungs at day 7 post surgery. Arrowheads denote regenerative MFs in either the ductal (yellow) or alveolar region (green). Airways were denoted by dashed white lines. Scale bars, 20 μm.

(P) Maximum intensity projection of ACTA2 and AGER staining at day 7 post PNX. Scale bars, 50 μm.

(Q) Schematic summary of cellular origins of regenerative MF.

(R-S) Representative immunofluorescent labeling of WT1 at the lung pleura from sham and PNX at 7 days post surgery. Quantifications were shown in (S). n=4 for each group. Scale bars, 20 μm.

(T) Trichrome staining in the lung at day 21 post PNX.

(U-V) Representative immunofluorescent labeling of MKI67 at the lung pleura from sham and PNX at 7 days post surgery. Quantifications were shown in (V). n=3 for each group. Scale bars, 20 μm.

(W) Profile of *Ebf2* expression on the integrated UMAP of mesenchymal cells, as presented by feature plot.

(X) Profile of *Wt1* expression on the UMAPs of mesenchymal cells separated by sham and PNX, as presented by feature plot.

(Y) Profile of *Aldh1a3* expression on the integrated UMAP of mesenchymal cells, as presented by feature plot.

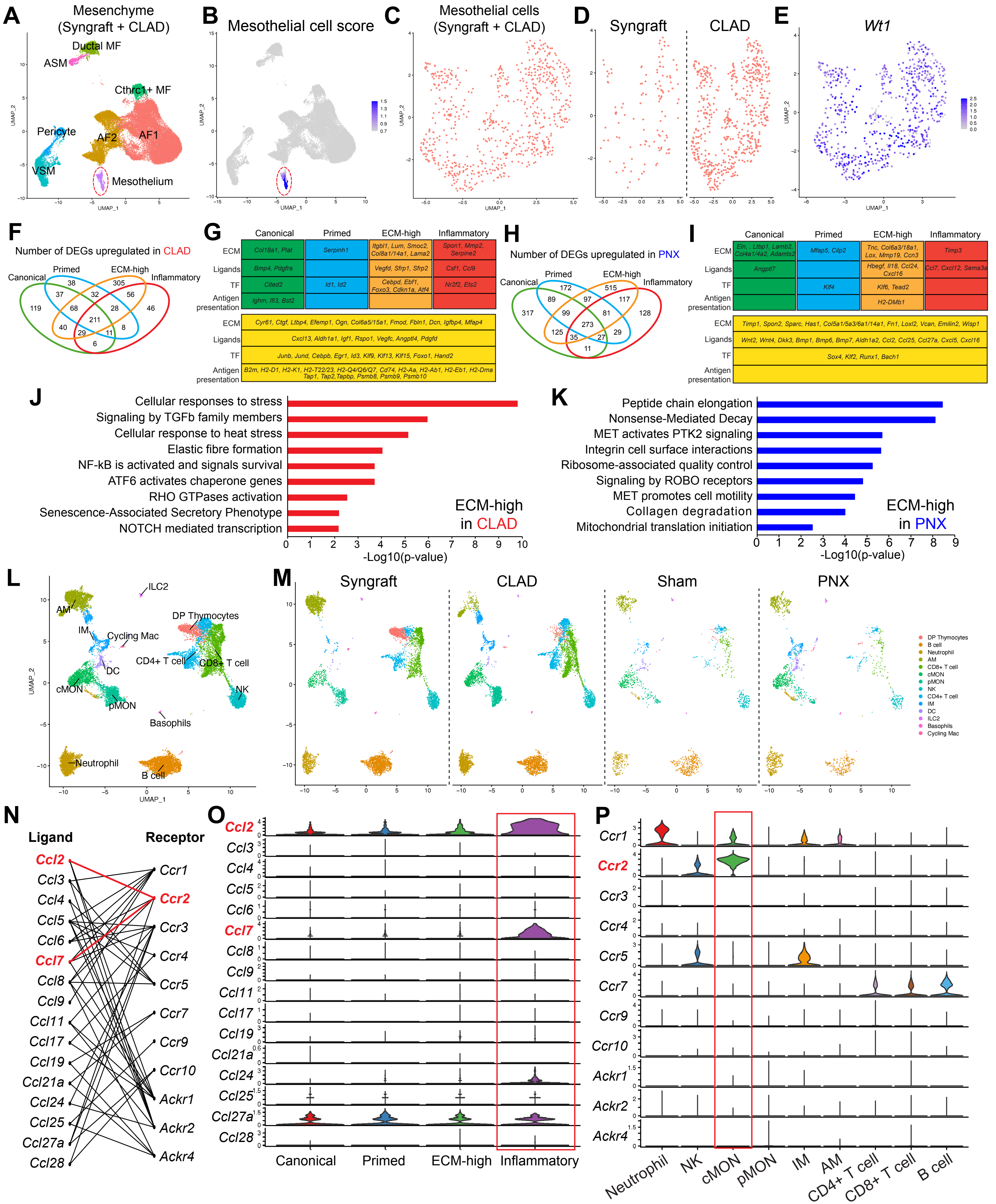
A
Mesenchyme (Syngraft + CLAD)
Ductal MF
ASM
Cthrc1+ MF
Pericyte
AF2
AF1
VSM
Mesothelium
B
Mesothelial cell score
C
Mesothelial cells (Syngraft + CLAD)
D
Syngraft
CLAD
E
Wt1
F
Number of DEGs upregulated in CLAD
Canonical
Primed
ECM-high
Inflammatory
G
Canonical
Primed
ECM-high
Inflammatory
ECM
Ligands
TF
Antigen presentation
Col18a1, Plat
Serpinh1
Itgbl1, Lum, Smoc2, Col8a1/14a1, Lama2
Spon1, Mmp2, Serpine2
Bmp4, Pdgfra
Vegfd, Sfrp1, Sfrp2
Csf1, Ccl9
Cited2
Id1, Id2
Cebpd, Ebf1, Foxo3, Cdkn1a, Atf4
Nr2f2, Ets2
Ighm, Ifi3, Bst2
Cyr61, Ctgf, Ltbp4, Efemp1, Ogn, Col6a5/15a1, Fmod, Fbln1, Dcn, Igfbp4, Mfap4
Cxcl13, Aldh1a1, Igf1, Rspo1, Vegfc, Angptl4, Pdgfd
Junb, Jund, Cebpb, Egr1, Id3, Klf9, Klf13, Klf15, Foxo1, Hand2
B2m, H2-D1, H2-K1, H2-T22/23, H2-Q4/Q6/Q7, Cd74, H2-Aa, H2-Ab1, H2-Eb1, H2-Dma Tap1, Tap2,Tapbp, Psmb8, Psmb9, Psmb10
H
Number of DEGs upregulated in PNX
Canonical
Primed
ECM-high
Inflammatory
I
Canonical
Primed
ECM-high
Inflammatory
ECM
Ligands
TF
Antigen presentation
Eln, , Ltbp1, Lamb2, Col4a1/4a2, Adamts2
Mfap5, Cilp2
Tnc, Col6a3/18a1, Lox, Mmp19, Ccn3
Timp3
Angptl7
Hbegf, Il18, Ccl24, Cxcl16
Ccl7, Cxcl12, Sema3a
Klf4
Klf6, Tead2
H2-DMb1
Timp1, Spon2, Sparc, Has1, Col5a1/5a3/6a1/14a1, Fn1, Loxl2, Vcan, Emilin2, Wisp1
Wnt2, Wnt4, Dkk3, Bmp1, Bmp6, Bmp7, Aldh1a2, Ccl2, Ccl25, Ccl27a, Cxcl5, Cxcl16
Sox4, Klf2, Runx1, Bach1
J
Cellular responses to stress
Signaling by TGFb family members
Cellular response to heat stress
Elastic fibre formation
NF-kB is activated and signals survival
ATF6 activates chaperone genes
RHO GTPases activation
Senescence-Associated Secretory Phenotype
NOTCH mediated transcription
ECM-high in CLAD
-Log10(p-value)
K
Peptide chain elongation
Nonsense-Mediated Decay
MET activates PTK2 signaling
Integrin cell surface interactions
Ribosome-associated quality control
Signaling by ROBO receptors
MET promotes cell motility
Collagen degradation
Mitochondrial translation initiation
ECM-high in PNX
-Log10(p-value)
L
ILC2
AM
DP Thymocytes
IM
Cycling Mac
CD4+ T cell
CD8+ T cell
DC
cMON
NK
pMON
Basophils
Neutrophil
B cell
M
Syngraft
CLAD
Sham
PNX
DP Thymocytes
B cell
Neutrophil
AM
CD8+ T cell
cMON
pMON
NK
CD4+ T cell
IM
DC
ILC2
Basophils
Cycling Mac
N
Ligand
Receptor
Ccl2
Ccl3
Ccl4
Ccl5
Ccl6
Ccl7
Ccl8
Ccl9
Ccl11
Ccl17
Ccl19
Ccl21a
Ccl24
Ccl25
Ccl27a
Ccl28
Ccr1
Ccr2
Ccr3
Ccr4
Ccr5
Ccr7
Ccr9
Ccr10
Ackr1
Ackr2
Ackr4
O
Canonical
Primed
ECM-high
Inflammatory
P
Ccr1
Ccr2
Ccr3
Ccr4
Ccr5
Ccr7
Ccr9
Ccr10
Ackr1
Ackr2
Ackr4
Neutrophil
NK
cMON
pMON
IM
AM
CD4+ T cell
CD8+ T cell
B cell

Figure S2. PNX-induced inflammatory mesothelial cells crosstalk with myeloid cells. Related to Figure 2.

(A) UMAP embedding of integrated mesenchymal cells from syngraft and CLAD lungs. Mesothelial cells are highlighted by the red dashed circle.

(B) Profile of module score of mesothelial cells on the integrated UMAP by feature plot.

(C-D) UMAP-based reclustering of integrated mesothelial cells from syngraft and CLAD lungs. Cells from individual conditions were separately shown in (D).

(E) Profile of *Wt1* expression on the integrated UMAP by feature plot.

(F, H) Venn diagrams showing the number of upregulated genes uniquely present or shared in each of the subtypes of mesothelial cells in CLAD (F) or PNX (H).

(G, I) Representative genes uniquely present or shared in each of the subtypes of mesothelial cells in CLAD (G) or PNX (I). DEGs shared by at least two subtypes are listed in the yellow boxes.

(J-K) Gene ontology analysis of DEGs in ECM-high mesothelial cells between CLAD in (J) versus PNX in (K).

(L-M) UMAP embeddings of integrated immune cells from syngraft, CLAD, sham and PNX lungs. Cells from different conditions were separately shown in (M).

(N) Ligand-receptor pairs in the CCL family that are expressed among interactions between inflammatory mesothelial cells and immune cells.

(O-P) Gene expression profiling of CCL2 ligands in expanded mesothelial cells (O) and receptors in immune cells (P), as presented by violin plots.

Figure S3 Xu et al.

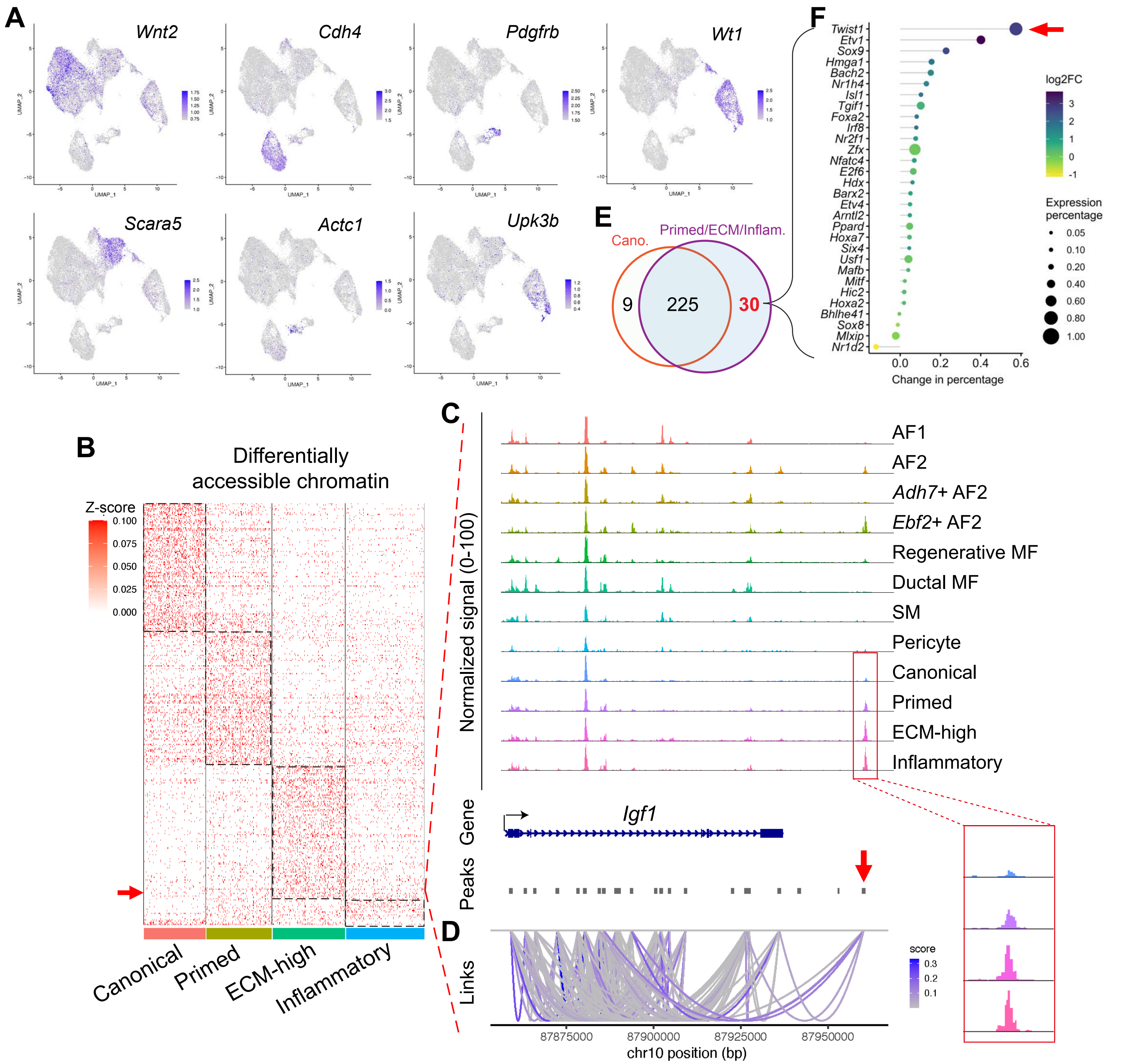

Figure S3. Characterization of scATAC-seq. Related to Figure 3.

(A) Expression profiling of marker genes of each cell type, as presented by feature plots.

(B) Differentially accessible chromatin of each cell subgroup in the expanded mesothelial cells.

(C-D) Chromatin accessibility dynamics of *Igf1* in the mesenchymal cells, as presented by coverage plots. Cis-regulatory DNA element interactions predicted by Cicero were shown (D).

(E) Number of enriched TFs from the predicted GRNs in canonical and activated mesothelial cells.

(F) A ranking of TFs in (E), which are exclusively enriched in activated mesothelial cells, based on their relative gene expression changes. Values of x-axis are calculated by subtracting gene expression percentage in canonical mesothelial cells from the gene's percentage in activated mesothelial cells.

Figure S4 Xu et al.

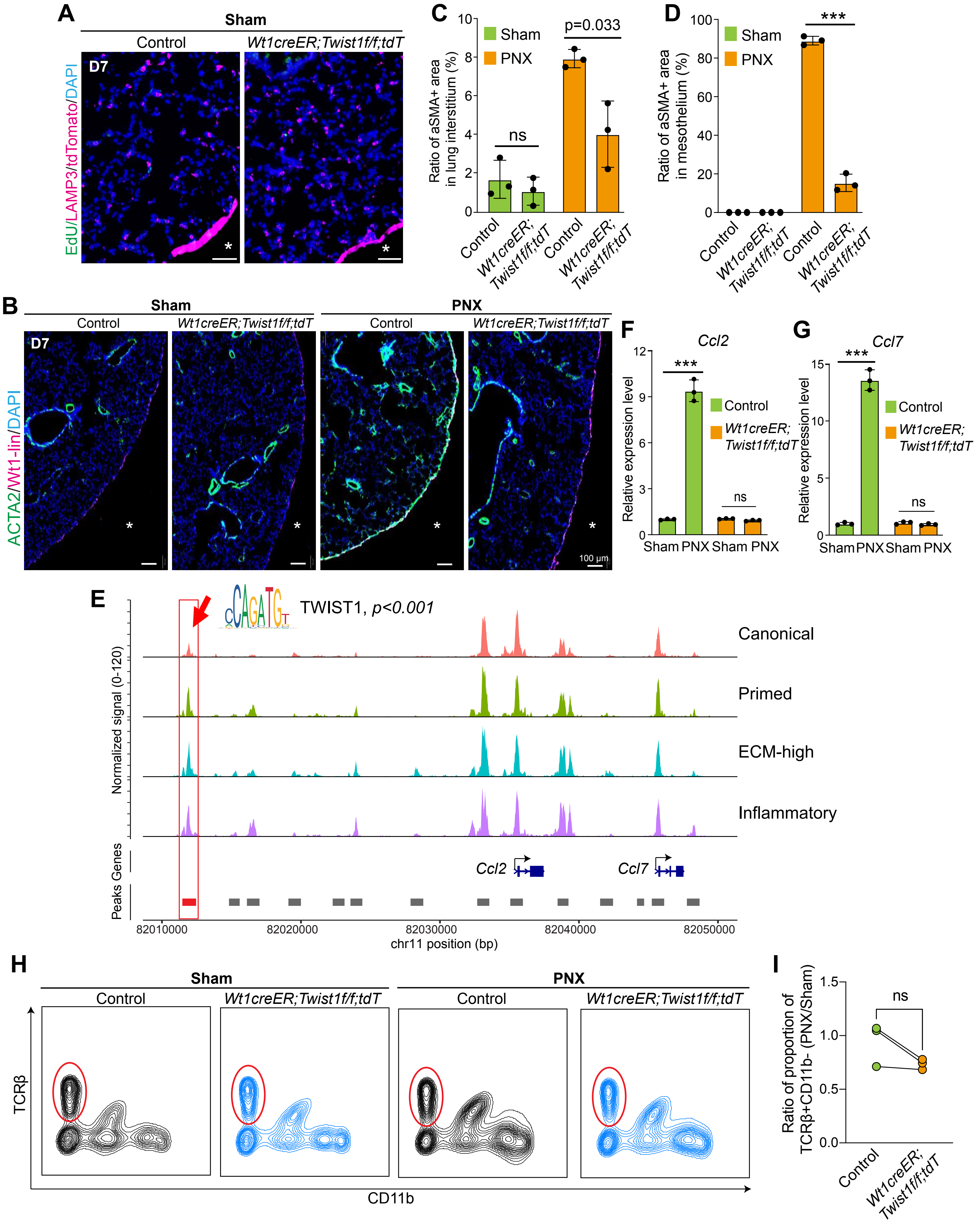

Figure S4. TWIST1 is required for mesothelial expansion. Related to Figure 4.

(A) Representative immunofluorescent labeling of EdU signals on top of LAMP3 in the lung of *Wt1creER;Twist1;tdT* mutants and relative controls at 7 days post sham. Scale bars, 20 μm.

(B-D) Representative immunofluorescent labeling of ACTA2 with *Wt1* lineage marker tdTomato from the lung of *Wt1creER;Twist1;tdT* mutants and relative controls at day 7 post surgery. Quantifications of ACTA2 staining in the interstitium and pleura were shown in (C) and (D), respectively. n=3 for each group. Scale bars, 20 μm.

(E) Track view of ATAC-seq signal around *Ccl2* and *Ccl7*. The boxed region highlights the DNA regulatory element predicted to be bound by TWIST1 (JASPAR motif MA1123.2).

(F-G) Quantification of *Ccl2* (F) and *Ccl7* (G) in the lung of *Wt1creER;Twist1;tdT* mutants and relative controls at day 7 post surgery, as assayed by RT-qPCR. n=3 for each group.

(H-I) Flow cytometry analysis of TCRβ+CD11b- T cells as gated in live CD45+ singlet in the lung of *Wt1creER;Twist1;tdT* mutants and littermate controls at 7 days post surgery. Quantifications within littermates were shown in (I). n=3 for each group.

Figure S5 Xu et al.

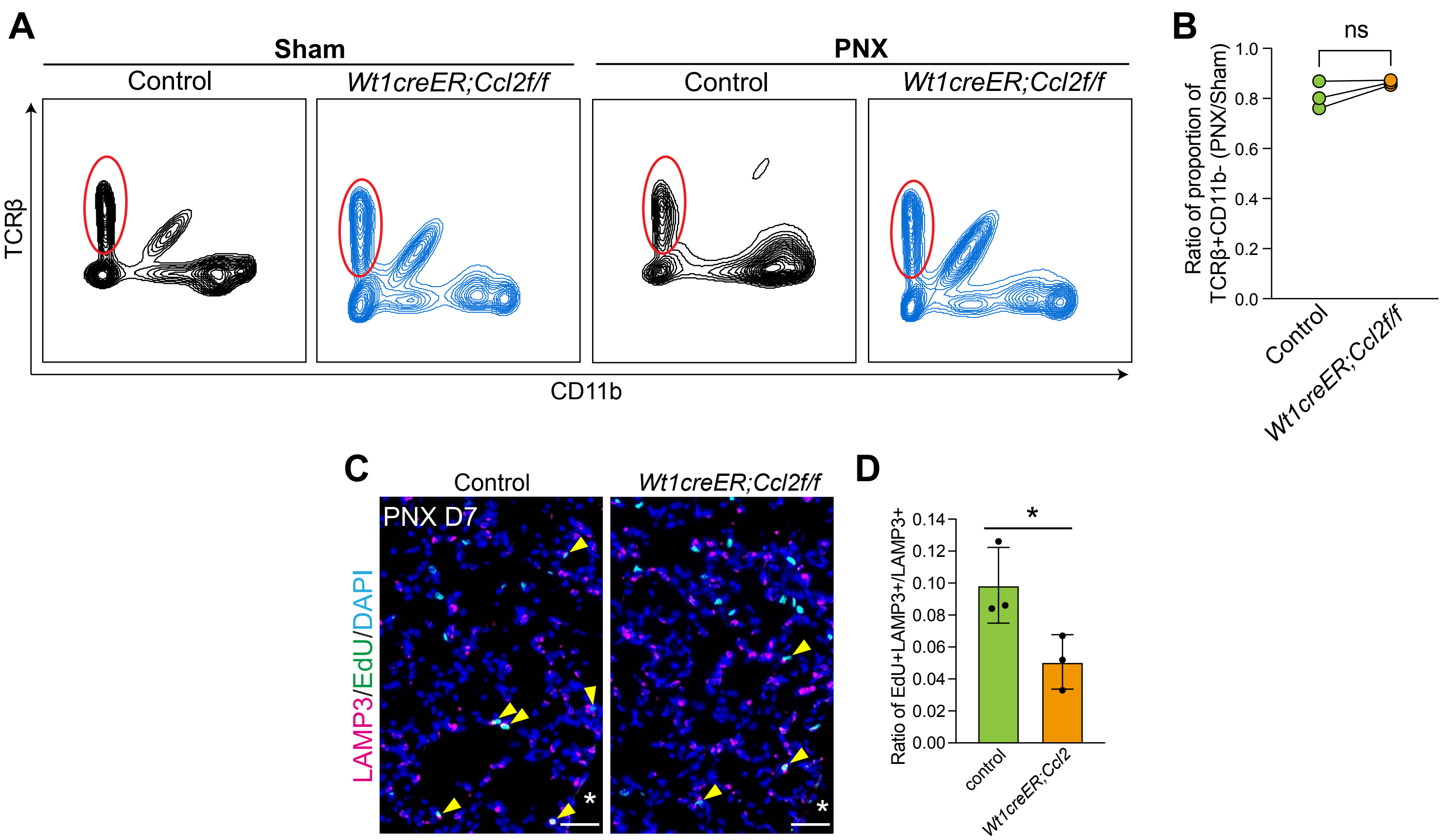

Figure S5. Mesothelial cell-derived CCL2 is required for AT2 proliferation post PNX. Related to Figure 5.

(A-B) Flow cytometry analysis of TCRβ+CD11b- T cells as gated in live CD45+ singlet in the lung of *Wt1creER;Ccl2* mutants and littermate controls at 7 days post surgery. Quantifications within littermates were shown in (B). n=3 for each group.

(C-D) Representative immunofluorescent labeling of EdU signals on top of LAMP3 in the lung of *Wt1creER;Ccl2* mutants and relative controls at day 7 post PNX. Quantifications were shown in (D). n=3 for each group. Scale bars, 20 μm.

Table S1. Lung samples of Sham/PNX for scRNA-seq and scATAC-seq. Related to Figures 1-5, S1-S and S4.

Strategy	Treatment	Sample ID	Tissue	Compartment
**scRNA-seq**	Sham	RNA_Sham-1	Right lobes	All
		RNA_Sham-2	Accessory lobe	Mesenchyme
	PNX D7	RNA_PNX-1	Right lobes	All
		RNA_PNX-2	Accessory lobe	Mesenchyme
**scATAC-seq**	Control	ATAC_Control (PMID:39910313)	All lobes	Mesenchyme
	PNX D7	ATAC_PNX	Accessory lobe	Mesenchyme

Table S2. Primer sequences used for qPCR. Related to Figures 1, 4, 5 and S4.

Gene	Sequence
Wt1	5'- GGTTTTCTCGCTCAGACCAGCT-3'
	5'- ATGAGTCCTGGTGTGGGTCTTC-3'
Ccl2	5'- GCTACAAGAGGATCACCAGCAG-3'
	5'- GTCTGGACCCATTCCTTCTTGG-3'
Ccl7	5'- CAGAAGGATCACCAGTAGTCGG-3'
	5'- ATAGCCTCCTCGACCCACTTCT-3'
Gapdh	5'- CATCACTGCCACCCAGAAGACTG-3'
	5'- ATGCCAGTGAGCTTCCCGTTCAG-3'
